# Deletion of Jun Proteins in Adult Oligodendrocytes Does Not Perturb Cell Survival, or Myelin Maintenance *In Vivo*

**DOI:** 10.1371/journal.pone.0120454

**Published:** 2015-03-16

**Authors:** Bettina Schreiner, Barbara Ingold-Heppner, Debora Pehl, Giuseppe Locatelli, Helia Berrit-Schönthaler, Burkhard Becher

**Affiliations:** 1 Institute of Experimental Immunology, University Zürich, Zürich, Switzerland; 2 Department of Neurology, University Hospital Zürich, Zürich, Switzerland; 3 Institute of Pathology, Charité—Universitätsmedizin Berlin, Berlin, Germany; 4 Institute of Neuropathology, Charité—Universitätsmedizin Berlin, Berlin, Germany; 5 Institute of Clinical Neuroimmunology, LMU Universität München, Germany; 6 Spanish National Cancer Research Center, Madrid, Spain; University Medical Center of the Johannes Gutenberg University of Mainz, GERMANY

## Abstract

Oligodendrocytes, the myelin-forming glial cells of the central nervous system (CNS), are fundamental players in rapid impulse conduction and normal axonal functions. JunB and c-Jun are DNA-binding components of the AP-1 transcription factor, which is known to regulate different processes such as proliferation, differentiation, stress responses and death in several cell types, including cultured oligodendrocyte/lineage cells. By selectively inactivating *Jun B* and *c-Jun* in myelinating oligodendrocytes *in vivo*, we generated mutant mice that developed normally, and within more than 12 months showed normal ageing and survival rates. In the adult CNS, absence of JunB and c-Jun from mature oligodendrocytes caused low-grade glial activation without overt signs of demyelination or secondary leukocyte infiltration into the brain. Even after exposure to toxic or autoimmune oligodendrocyte insults, signs of altered oligodendrocyte viability were mild and detectable only upon cuprizone treatment. We conclude that JunB and c-Jun expression in post-mitotic oligodendrocytes is mostly dispensable for the maintainance of white matter tracts throughout adult life, even under demyelinating conditions.

## Introduction

The transcription factors c-Jun and JunB are key components of the activator protein-1 (AP-1) transcription factor complex and form, together with JunD, the Jun protein family (reviewed in [1,2]). N-terminal phosphorylation of c-Jun by c-Jun N-terminal kinases (JNK) can alter AP-1 binding activity in the absence of *de novo* protein synthesis, but c-Jun has also phosphorylation-independent functions [3,4,5]. Proteins of the Jun family are critical regulators of multiple cellular processes including differentiation, proliferation, and apoptosis, often with opposing outcomes depending on the cellular context (reviewed in [6]). Germ-line deletion of *c-Jun* leads to embryonic lethality [7,8]. However, *JunB* ^*flox/flox*^ [9] and *c-Jun* ^*flox/flox*^ [10] mice have been used to selectively inactivate Jun proteins in various cell types and tissues, including skin (*K5-cre-ERT*; [11]), liver (*albumin-cre/Mx-cre*; [10]), peripheral nerve glia (*P*_*0*_-*cre*; [12,13]) and nervous tissue (early neuroepithelial *nestin-cre*; [14]; neuronal *syn-cre*; [5]). This transgenic cre-lox approach thus allowed to describe some of the fundamental roles of Jun proteins in several pathological conditions, as in psoriatic skin lesions in which epidermal keratinocytes show decreased expression of JunB, and inducible epidermal deletion of JunB and c-Jun causes a fulminant psoriasis-like skin disease and arthritis in mice [11].

c-Jun expression in glial Schwann cells plays a critical role in repair responses after nerve injury in the peripheral nervous system (PNS), which is known to have a high regenerative capacity compared to the CNS [12,13]. Deletion of *c-Jun* using a Cre-recombinase driven by the nestin promotor decreases efficient axonal regeneration after transsection of the facial nerve [14], and selective inactivation of *c-Jun* in Schwann cells impairs axon re-growth and nerve target re-innervation after injury, as well as myelin clearance by macrophages. Despite these injury-related functions of c-Jun, absence of the gene did not affect normal Schwann cell and nerve functions in adult uninjured mice [13].

In contrast to the PNS, the final effect of Jun proteins on oligodendroglial fate in the CNS remains controversial. While some investigators show that induction of c-Jun by nerve growth factor or tumor necrosis factor (TNF) in oligodendrocytes correlates with apoptosis *in vitro* [15,16], others report activation of JNK without apoptosis by TNF in astrocyte and oligodendrocyte cultures [17]. In active multiple sclerosis (MS) lesions, up-regulation of nuclear staining for c-Jun/JNK proteins on a large proportion of oligodendrocytes located at the edge of active lesions has been described [18]. The concomitant absence of oligodendroglial cell death would speak against a direct role of c-Jun in the apoptotic process of these glial cells.

To further elucidate the function of AP-1 proteins in oligodendrocyte biology in the adult CNS *in vivo*, we used mutants with oligodendrocyte-specific deletion of *JunB* and *c-Jun* (at late myelinating stages in these cells). We examined the role of these factors in the uninjured CNS, and after inducing oligodendrocyte damage by mitochondrial impairment [19] following cuprizone application and induction of myelin-directed autoimmunity. Our study indicates, that oligodendroglial JunB and c-Jun have at the most a minor protective effect on oligodendrocyte survival and myelination, even upon demyelinating insults. Nevertheless, our data do underscore the tissue- and context-dependent differences in Jun protein function *in vivo*, and the fact that they often can only incompletely be predicted by *in vitro* studies on primary/lineage cells.

## Materials and Methods

### Mice and genotyping

All animal experiments were specifically approved by the Institutional Animal Care and Use Committee and Swiss Cantonal Veterinary Office (License 86/2012, Zurich, Switzerland). Mice carrying a *floxed JunB* allele (*junB* ^*f/f*^; [9]) and/or *floxed c-Jun* allele (*c-jun* ^*f/f*^; [10]) were crossed to transgenic mice animals expressing the Cre recombinase under the control of the oligodendrocyte-specific MOG promoter (*MOGi-cre*; [20]) to obtain *JunB* ^*f/f*^ *c-Jun* ^*f/f*^ *MOGi-cre* mice (*JunB* ^*Δol*^/*c-Jun* ^*Δol*^ double mutants). Sibling animals lacking the Cre transgene, with functional, unrecombined homozygous *JunB* and *c-Jun* (*JunB* ^*f/f*^/*c-Jun* ^*f/f*^), served as controls. CO_2_ inhalation was used as method of euthanasia.

The primer sequences for genotyping were: *MOGi-cre* (WT 350 bp): GAC AAT TCA GAG TGA TAG GAC CAG GGT ATC CC and GCT GCC TAT TAT TGG TAA GAG TGG; *MOGi-cre* (knock-in, 700 bp): TCC AAT TTA CTG ACC GTA CAC and CAT CAG CTA CAC CAG AGA CGG AAA TC; *JunB* (WT 299 bp, floxed 384 bp): ATC CTG CTG GGA GCG GGG AAC TGA GGG AGG and AGA GTC GTC GTG ATA GAA AGG C; *JunB* (WT 1490 bp, floxed 1575 bp, Δ 300 bp): GGG AAC TGA GGG AAG CCA CGC CGA GAA AGC and AAA CAT ACA AAA TAC GCT GG; *c-Jun* (WT 300 bp, floxed 350 bp, Δ 600 bp): CAG GGC GTT GTG TCA CTG AGC T and CTC ATA CCA GTT CGC ACA GGC GGC and CCG CTA GCA CTC ACG TTG GTA GGC.

### Western Blot analysis

CNS tissues were lysed in cell lysis buffer (Cell Signaling), supplemented with cOmplete Protease Inhibitor Cocktail and PhosSTOP Phospatase inhibitors (both Roche), for 30 min, sonicated and centrifuged at 14`000 x *g* at 4°C for 30 min. After BCA assay (Thermo Scientific), proteins were blotted and detected with the following antibodies: mouse anti-Vinculin (loading control, 1: 20`000, clone hVIN-1, Sigma), rabbit polyclonal anti-junB (1:250, Santa Cruz, sc-46) and mouse anti-c-jun (1:1`000, clone 3/Jun, BD Transduction Laboratories).

### Scoring of motor performance

In RotaRod experiments, the average time to fall was measured during a 5–50 rpm acceleration over 3 min (*n* = 3). In the walking grid test, we counted the number of footfalls over a 50-cm-long runway with irregularly arranged bars (0.5–2.5 cm) on a 10-cm distance. Mice were assigned EAE scores daily as follows: 0, no detectable signs of EAE; 0.5, distal tail limp; 1, complete tail limp; 2, unilateral partial hindlimb paralysis; 2.5, bilateral partial limb paralysis; 3, complete bilateral hindlimb paralysis; 3.5, complete hindlimb paralysis and unilateral forelimb paralysis; 4, total paralysis of forelimbs and hindlimbs (mice with a score above 4 to be euthanized); 5, death.

### Immunohistochemistry

CNS was perfused, fixed (4% FA in PBS) and after paraffin-embedding cut at 5 μm. Hematoxylin and eosin and LFB-PAS staining were performed according to standard protocols. CNPase (mouse, clone 11-5B, Chemicon/Millipore), polyclonal Iba-1 (rabbit, Wako) and polyclonal GFAP (rabbit, DAKO) staining was performed by a Ventana Benchmark XT-automated staining according to the manufacturer's guidelines (iVIEW DAB Detection Kit, Ventana).

In order to evaluate demyelination, LFB-PAS stained brain sections of cuprizone treated mice were scored in a blinded fashion from zero to three as described before [21]. Zero was equivalent to the myelin status of a mouse not treated with cuprizone, whereas a score of three was total demyelination of the corpus callosum. A score of 1 is equivalent to demyelination of one third of the fibers, while a score of two is equivalent to demyelination of two thirds of the fibers of the myelin tract. Iba-1^+^ microglial cells were counted manually in a blinded fashion. Only cells that contained a nucleus, as indicated by hematoxylin counterstain, were counted. Four sections per animal were analyzed and values averaged per mouse. EAE spinal cord sections were semi-quantitatively analyzed, assessing inflammation (H/E, lymphocytes: 0: none, 1: mild, 2: moderate, 3: strong) and demyelination (LFB-PAS and CNPase: 0: none, 1: mild, 2: moderate, 3: strong).

### CNS flow cytometry

Mice were perfused using ice-cold PBS and brainstem with cerebellum and spinal cords were collected. Tissues were cut into small pieces using scissors, followed by 30 min of digestion with 0.8 mg/mL collagenase D (Roche) and 0.5 mg/mL DNAse (Sigma). Remaining pieces of tissue were homogenized and filtered through a 100 μm-cell strainer. After washing, the cell suspension was loaded onto a continuous 30% Percoll (GE) gradient and centrifuged for 30 min at 15 000 × *g*. The myelin layer was removed carefully, and the remaining cell suspension spun down. Flow cytometric analysis was performed following standard methods. We purchased 30-F11 (CD45) and M1/70 (CD11b) antibodies from BD Biosciences. In all stainings, dead cells were excluded using an Aqua Live/Dead fixable staining reagent (Invitrogen), and absolute cell numbers were determinded using AccuCheck Counting Beads (Life Technologies).

### Cuprizone Treatment

Eight to ten week old male mice were fed 0.2% to 0.4% wt/wt cuprizone (bis(cyclohexylidenehydrazide), C9012-25G, Sigma) to induce demyelination [22].

### EAE induction

For EAE experiments 6 to 10 weeks old female mice were immunized subcutaneously with 200 μg (each flank 100 μg) of MOG_35–55_ peptide (MEVGWYRSPFSRVVHLYRNGK) emulsified in Complete Freund’s Adjuvant (CFA, H37 Ra, Difco laboratories), and injected i.p. the same day and at day 2 with 200 ng pertussis toxin (Sigma).

### Statistical analysis

Results are given as mean ± s.e.m. unless indicated otherwise. Statistical significance was determined with GraphPad Prism (GraphPad Software).

## Results and Discussion

### Generation of JunB^Δol^/c-Jun^Δol^ mice mice lacking Jun proteins in mature oligodendrocytes

**Fig 1:**
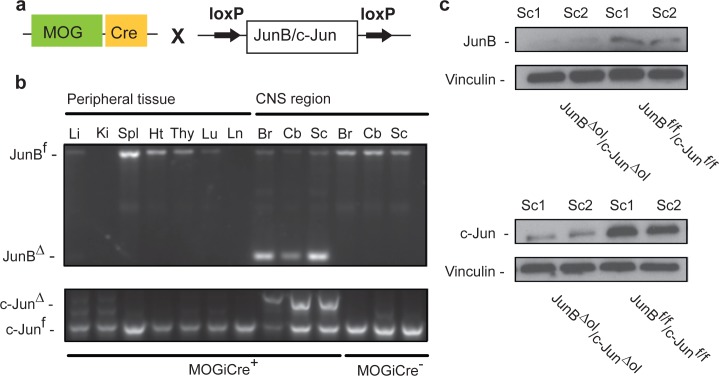
Deletion of JunB and c-Jun in oligodendrocytes. (a) Schematic diagram of MOGiCre x JunB^f/f^ and c-Jun^f/f^ (JunB^Δol^/Jun-c^Δol^) mice. (b) Genotyping PCR using genomic DNA derived from various peripheral organs and CNS regions from a JunB^flox/flox^ (top) and c-Jun^flox/flox^ (bottom) MOGiCre/+ mutant (JunB^Δol^/c-Jun^Δol^) and from a homozygous JunB^flox/flox^/c-Jun^flox/flox^ MOGiCre-negative control (JunB^f/f^/c-Jun^f/f^). Li, liver; Ki, kidney, Spl, spleen; Hr, heart; Thy, thymus, Lu, lung; Ln, lymph node; Br, brain; Cb, cerebellum; Sc, spinal cord. (c) Western Blot analysis for JunB, c-Jun and vinculin (loading control) in protein lysates obtained from CNS spinal cord tissue of junB^f/f^c-Jun^f/f^ controls and JunB^Δol^/c-Jun^Δol^ double mutants (n = 2 individual animals per group).

We aimed at studying the functions of *JunB* and *c-Jun* in mature oligodendrocytes in an *in vivo* mouse model. Therefore, we generated animals with an oligodendrocyte specific deletion of the *JunB* and *c-Jun* gene by crossing mice carrying LoxP-site-containing (*floxed*) *JunB* (*JunB* ^*f/f*^) and *c-Jun* (*c-Jun* ^*f/f*^) alleles with animals expressing the Cre recombinase under the control of the myelin oligodendrocyte glycoprotein (MOG) promoter (Fig. 1A). As MOG is the last of myelin proteins to be produced along oligodendrocyte maturation [23,24], genetic recombination in this *MOGi-cre* strain is specific for terminally differentiated oligodendrocytes [25]. Previous studies have demonstrated the CNS specifity and efficiency of the *MOGi-cre* transgenic line [20]. Because Cre/LoxP-mediated recombination can vary between different loxP-flanked target genes, we analyzed genomic DNA of various organs of adult *MOGi-cre* ^*+/-*^ *JunB* ^*f/f*^/*c-Jun* ^*f/f*^ (*JunB* ^*Δol*^/*c-Jun* ^*Δol*^) mice by PCR. A 300- or 600-bp product corresponding to the deleted/recombined *JunB* or *c-Jun* gene was observed solely in DNA taken from CNS tissues, thus confirming CNS-specific recombination in this mouse strain (Fig. 1B). Significant *JunB* ^*f*^ or *c-Jun* ^*f*^ inactivation was not confined to a specific CNS region, as it was observed in brain (Br), cerebellum (Cb) and spinal cord (Sc) (Fig. 1B). Furthermore, we tested the protein levels of JunB and c-Jun in CNS lysates from spinal cord of three to four months-old *JunB* ^*Δol*^/*c-Jun* ^*Δol*^ mice. We confirmed that the levels of JunB and c-Jun protein expressed in *JunB* ^*Δol*^/*c-Jun* ^*Δol*^ CNS spinal cord tissues were reduced compared to Cre-negative floxed control mice (Fig. 1C).

### Normal brain morphology and motor behaviour in JunB^Δol^/c-Jun^Δol^ mice

**Fig 2:**
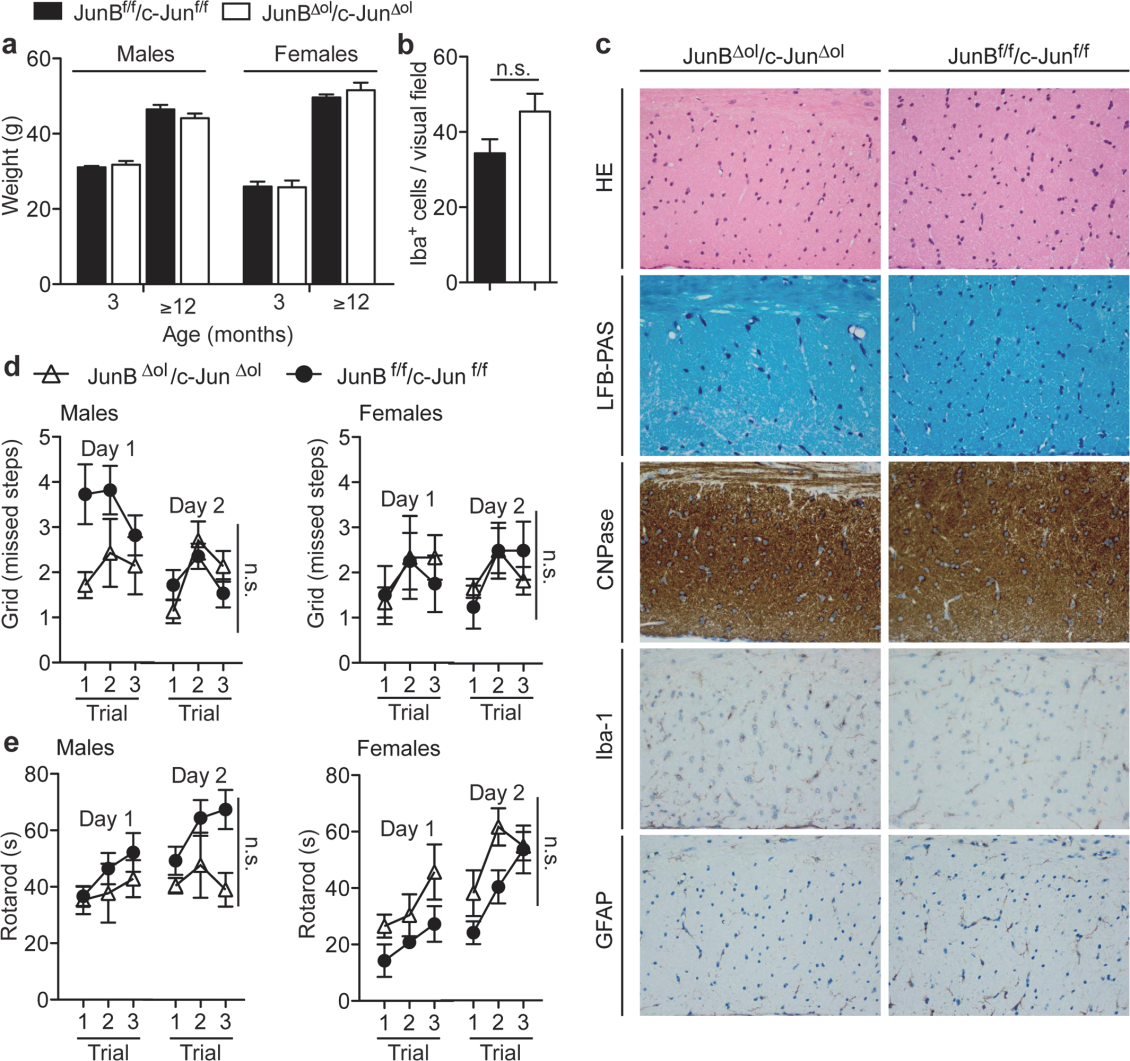
Normal brain morphology and motor skills. (a) Weight of littermate controls (JunB^f/f^c-Jun^f/f^) (black bars) and JunB^Δol^/c-Jun^Δol^ double mutants (white bars) at 3 and ≥12 months of age. 22 controls and 30 mutants were analyzed in total. (b, c) Histological analysis (hematoxylin and eosin, HE and luxol fast blue—periodic acid schiff staining, LFB-PAS) of control and mutant brain sections at 6–12 months of age (n≥4 per group; representative pictures are from the corpus callosum brain region of 6 month old mice). Immunostaining for oligodendroglial CNPase, microglial Iba-1, and astrocytic GFAP. Scale bar, 50 μm. For quantifications of the number of Iba-1^+^ cells per visual field see (b) (n = 3–5 mice per group, unpaired t-test, n.s., not significant). (d, e) The grid test (d) assessing limb strenght and subtle motor coordination deficits like slipping at ≥12 months of age (n = 15 controls, and n = 13 double mutants). Motor performance in the rotarod test (e) (3 consecutive trials; after 3 exercise trials the day before). Matched 2way ANOVA, Bonferroni post-test, n.s., not significant; *P < 0.05.

*MOGi-cre* ^*+/-*^ *JunB* ^*f/f*^/*c-Jun* ^*f/f*^ mice were born with Mendelian frequency (49% mutant pups compared with 50% expected, n = 151) and were viable and fertile. Males and females presented with normal general health, and body weight as compared to control mice at three months of age (male controls 31±0 vs mutants 32±1 g, p = 0.6082 and female controls 26±1 vs mutants 26±2 g; p = 0.9399) and up to more than 12 months postnatally (male controls 46±1 vs mutants 44±1 g, p = 0.2279 and female controls 50±1 vs mutants 52±2 g; p = 0.4738, unpaired, two-tailed t-test; Fig. 2A). *JunB* ^*Δol*^/*c-Jun* ^*Δol*^ mice showed similar CNS cyto- and myelo-architecture compared to controls (Fig. 2B and C) by H&E, LFB-PAS and myelin-associated CNPase staining at up to six to twelve months of age. We did not observe any apparent demyelination, even though there was a mild increase in the number of activated microglia (Iba-1 staining: controls 34.3±4 vs mutants 45.4±5 Iba^+^ cells/visual field, p = 0.1601, unpaired t-test, n = 3–5 mice; for quantifications see Fig. 2B) and mild reactive astrogliosis (GFAP) compared to Cre-negative floxed control mice. Notably, absence of *JunB* and *c-Jun* from mature oligodendrocytes did not cause infiltration of leukocytes into the brain (Fig. 2C, HE staining, and data not shown). Moreover, deletion of *JunB* and *c-Jun* in oligodendrocytes did not lead to obvious neurological deficits indicative of disturbed CNS myelin maintenance up to the age of more than one year (Fig. 2D and E; maximal observation time was 19 months of age). In order to detect more suble motor impairments, we challenged *JunB* ^*Δol*^/*c-Jun* ^*Δol*^ mice using a grid test (Fig. 2D). They showed similar missteps per trial compared to controls (on average over 3 trials: male controls 2±0 versus mutants 2±0; p = 0.0988 and female controls 2±0 versus mutants 2±0, p = 0.9306 for genotype, matched two-way ANOVA). When we subjected the animals to the rotarod testing, (Fig. 2E) motor performance was comparable in male and female *JunB* ^*Δol*^/*c-Jun* ^*Δol*^ mice compared to Cre-negative floxed controls. However, there were signs of slight motor learning deficits, in that male double mutants did improve their performance less during three consecutive trials (male controls improved 1.4-fold, mutants 0.9-fold, p = 0.0109; female controls improved 2.4-fold, mutants 1.8-fold, p = 0.3719, unpaired, two-tailed t-test; Fig. 2E). Thus, staining for astrocytes and microglia gave an indication of low-grade inflammation in elderly *JunB* ^*Δol*^/*c-Jun* ^*Δol*^ mutant mice, which is a very sensitive response to degenerative processes in white matter tracts. However, we did not observe more prominent signs of oligodendrocyte loss or demyelination by light microscopy criteria, nor clinically relevant motor deficits. Taken together, the post-myelination CNS phenotype of adult *JunB* ^*Δol*^/*c-Jun* ^*Δol*^ double mutants mice was mild, and oligodendrocytes seemed to be sufficiently able to maintain myelin.

### Comparable cuprizone-induced demyelination in JunB^Δol^/c-Jun^Δol^ and control mice

**Fig 3:**
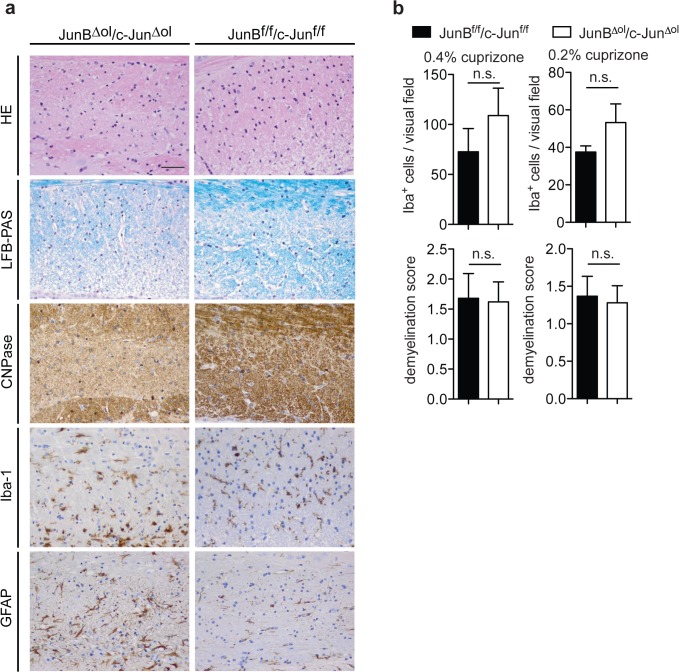
Comparable cuprizone-induced demyelination after oligodendroglial deletion of JunB and c-Jun. (a) Representative images of JunB^f/f^/c-Jun^f/f^ controls and JunB^Δol^/c-Jun^Δol^ double mutants that received cuprizone for 6 weeks. (b) The number of microglia and extent of demyelination was evaluated in matched sections (n = 3–5 mice per group, n = 4 sections per mouse averaged, unpaired t-test). HE, LFB-PAS staining, as well as CNPase, Iba-1 and GFAP immunoreactivity in the corpus callosum. Scale bar, 50 μm.

To investigate *JunB* and *c-Jun* function in mature oligodendrocytes in response to a toxic demyelinating insult, we fed eight to ten week old *JunB* ^*Δol*^/*c-Jun* ^*Δol*^ mice with cuprizone [26] (Fig. 3A and B). Cuprizone feeding damages oligodendrocytes progressively and dose-dependently. It leads to consistent oligodendrocyte cell loss starting three weeks post-administration (p.a.), accompanied by the first signs of demyelination [27]. We compared the effects of cuprizone feeding in *JunB* ^*Δol*^/*c-Jun* ^*Δol*^ double mutant and *JunB* ^*f/f*^/*c-Jun* ^*f/f*^ control mice at week six p.a., when the corpus callosum generally is maximal demyelinated. At that time point, most of our control mice showed robust and moderate to strong demyelination which affected one to two thirds of the corpus callosum (LFB-PAS and CNPase staining), activated microglia (Iba-1) and reactive GFAP-positive astrocytes. We did not detect significant leukocyte accumulation (HE staining). Demyelination was similar in *JunB* ^*Δol*^/*c-Jun* ^*Δol*^ double mutant and control mice (LFB-PAS/demyelination score: controls 1.3±0.3 vs mutants 1.2±0.2, p = 0.8176 for 0.2% cuprizone, and controls 1.7±0.4 vs mutants 1.6±0.7, p = 0.9126 for 0.4% cuprizone, unpaired t-test, n = 3–5 mice; for quantifications see Fig. 3B). Notably, in two to three experiments in which changes in control mice were more discrete (reactive astrocytes at week 4 p.a., no later demyelination or only moderate demyelination), we detected a minor increase in reactive gliosis and/or demyelination in *JunB* ^*Δol*^/*c-Jun* ^*Δol*^ double mutants (GFAP-upregulation starting at week 2 p.a., more pronounced microgliosis at later time points or more demyelination).

**Fig 4:**
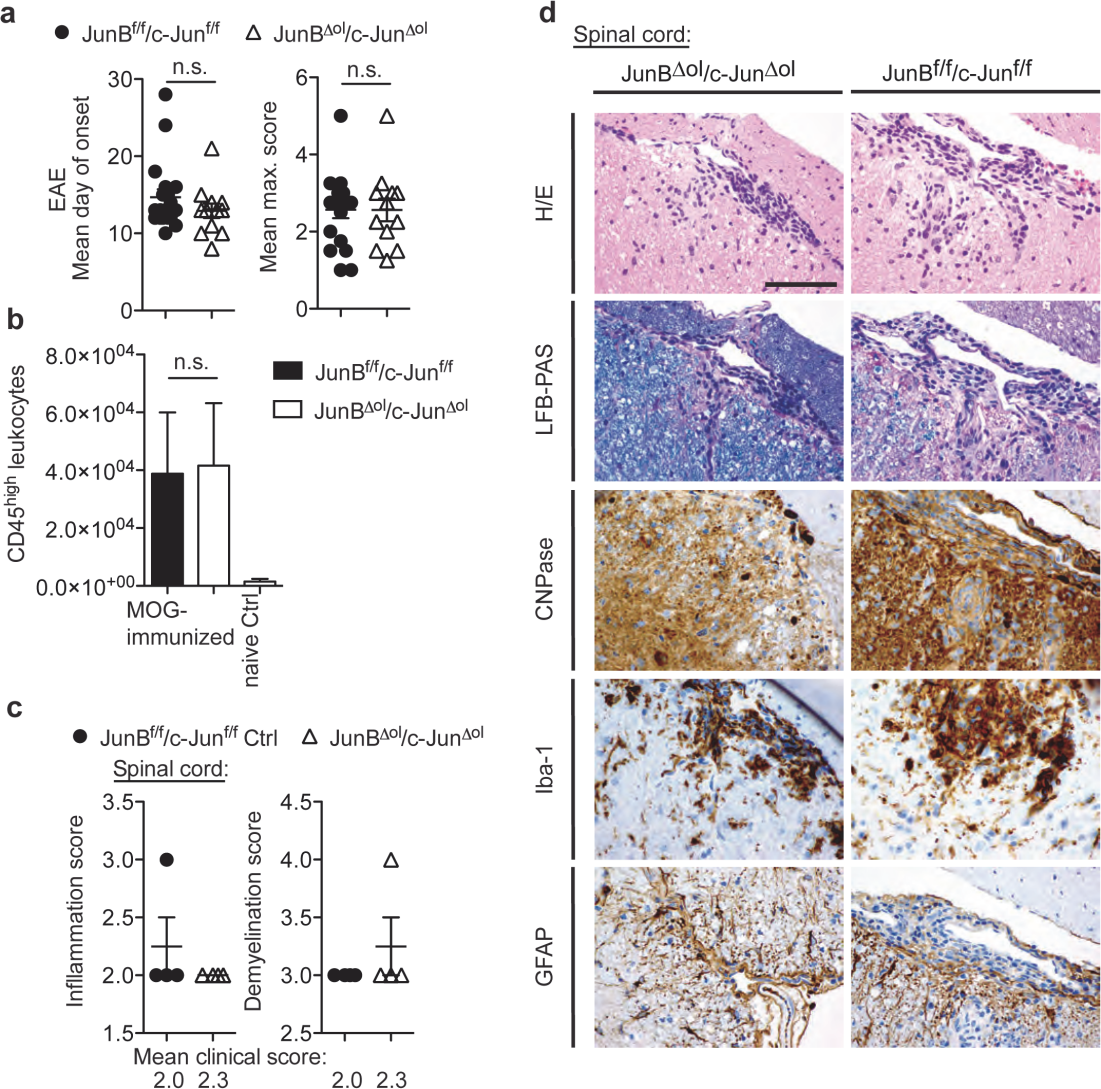
Similar clinical and histopathological EAE phenotype after oligodendroglial knock-out of JunB and c-Jun. (a) Day of onset and maximal clinical EAE score in MOG_35–55_-immunized control (JunB^f/f^c-Jun^f/f^) and JunB^Δol^/c-Jun^Δol^ double mutants (n = 12–19, unpaired t-test). (b) Number of CD45^high^ leukocytes isolated from cerebellum/spinal cords of controls and mutant mice. Data were pooled from 2 independent experiments (n = 10 MOG peptide-immunized mice per group and n = 4 naïve mutants, unpaired t-test). (c) Semiquantitative histology scores of inflammation and demyelination in spinal cords of MOG peptide-immunized mutant mice (mean clinical EAE score 2.3) and controls (mean EAE score 2.0; n = 4 per group). (d) Representative images of HE, LFB-PAS staining, as well as CNPase, Iba-1 and GFAP immunoreactivity in the spinal cords.

In addition, we challenged oligodendrocytes in *JunB* ^*Δol*^/*c-Jun* ^*Δol*^ CNS by triggering a T cell-mediated immune attack against CNS myelin (MOG_35–55_ peptide-induced Experimental Autoimmune Encephalomyelitis, EAE) (Fig. 4). In this mouse model for MS, demyelination and cell death is observed to a variable degree predominantly in spinal cord and brain stem [28]. In diseased mice, the day of onset of neurological deficits (n = 12–19, p = 0.2478, unpaired t-test) and maximal clinical score (p = 0.9928) were similar in *JunB* ^*Δol*^/*c-Jun* ^*Δol*^ double mutant and control mice (Fig. 4A). We assessed leukocyte infiltration (by flow cytometry staining for CD45, Fig. 4B, and HE histochemistry, Fig. 4 C and D) and demyelination in EAE brains and spinal cords of mice with similar acute disease severities (by LFB-PAS and CNPase staining, n = 4 *JunB* ^*Δol*^/*c-Jun* ^*Δol*^ double mutants with an average clinical score of 2.3 and n = 4 controls with an average score of 2.0;). As expected, leukocyte infiltration was prominent in the spinal cords, but spinal demyelination comparably strong between double mutants and controls (Fig. 4C and D).

Even though both, toxic cuprizone treatment and EAE, have limitations as models of human chronic, slowly progressive demyelinating diseases, like MS (reviewed in [29]), the cuprizone model can still provide insights into the determinants of oligodendrocyte death *in vivo*, and EAE has proven very useful to study inflammatory aspects of MS. In this regard, this study also speaks against the hypothesis, that loss of c-Jun and JunB in the adult CNS would mirror what is observed in the skin, in that AP-1 dysfunction is not a strong universal trigger of inflammation. That the survival of mature oligodendrocytes after demyelinating toxic (cuprizone) or autoimmune insults (EAE) apparently is only slightly or not dependent on JunB and c-Jun activity *in vivo* was unexpected based on previous studies of cultured oligodendrocyte/lineage cells. In fact, oligodendrocyte/lineage cell proliferation and process extension *in vitro* seems to be impaired when overexpressing a dominant-negative c-Jun mutant [30]. In addition, JNK and AP-1 DNA-binding activity in cultured oligodendrocytes have been correlated with apoptosis induced by different stimuli [30,31,32,33,34]. However, when glial cells are maintained in tissue culture, their phenotype often changes considerably and they might not fully reflect all properties of myelinated, mature oligodendrocytes *in vivo* [30,35]. Our work shows in an *in vivo* model, that if JunB and c-Jun have any function in oligodendrocyte homeostasis, it must be a redundant one.

We conclude that once myelination has occurred, reduced JunB and c-Jun functions do not significantly perturb oligodendrocyte survival, or myelin maintenance *in vivo*.

## Supporting Information

S1 ARRIVE Checklist Completed “The ARRIVE Guidelines Checklist” for reporting animal data in this manuscript. (PDF)

S1 Fig Full scans of Western Blots. (EPS)
